# Optimisation of GraPhage13 macro-dispersibility *via* understanding the pH-dependent ionisation during self-assembly: towards the manufacture of graphene-based nanodevices†

**DOI:** 10.1039/d3nr00778b

**Published:** 2023-07-25

**Authors:** Kate Stokes, Yiwei Sun, Paolo Passaretti, Henry White, Pola Goldberg Oppenheimer

**Affiliations:** a School of Chemical Engineering, Advanced Nanomaterials Structures and Applications Laboratories, College of Engineering and Physical Sciences, University of Birmingham Edgbaston Birmingham B15 2TT UK GoldberP@bham.ac.uk; b Institute of Cancer and Genomic Sciences, College of Medical and Dental Sciences, University of Birmingham B15 2TT UK; c BAE-Systems Air Sector Buckingham House FPC 267 Filton Bristol UK; d Healthcare Technologies Institute, Institute of Translational Medicine Mindelsohn Way Birmingham B15 2TH UK

## Abstract

GraPhage13 aerogels (GPAs) are micro-porous structures generated through the self-assembly of graphene oxide (GO) and M13 bacteriophage. As GPA fabrication involves the aggregation of GO and M13 in aqueous solution, we aim to understand its dispersibility across a wide pH range. Herein, a novel technique has been developed to relate the ionisation of functional groups to the surface charge, offering insights into the conditions required for GPA fabrication and the mechanism behind its self-assembly. The aggregation of GO and M13 was observed between pH 2–6 and exhibited dependence on the surface charge of the resulting aggregate with the M13 bacteriophage identified as the primary factor contributing to this, whilst originating from the ionisation of its functional groups. In contrast, GO exhibited a lesser impact on the surface charge due to the deprotonation of its carboxylic, enolic and phenolic functional groups at pH 6 and above, which falls outside the required pH range for aggregation. These results enhance our understanding of the GPA self-assembly mechanism, the conditions required for their fabrication and the optimal processability, laying the foundation towards its broad range of applications and the subsequent manufacture of graphene-based nanodevices.

## Abstract 2

GraPhage13 aerogels (GPAs) are micro-porous structures generated through the self-assembly of graphene oxide (GO) and M13 bacteriophage.

## Graphical abstract

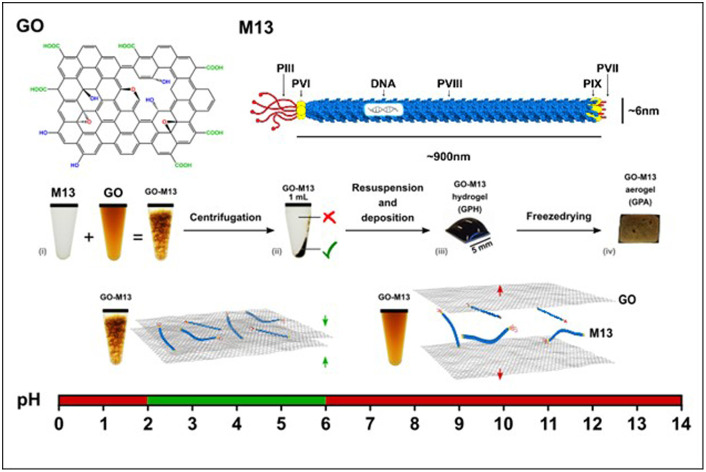

## 1. Introduction

Graphene and related nanomaterials have been attracting an exceptionally high research interest for almost two decades within the scientific community, due to their outstanding structural, optical, electrical, thermal and mechanical properties.^1,2^ To realise their full potential of impacting daily life, it is imperative to establish robust, straightforward, and scalable manufacturing methods for graphene-based nanomaterials, enabling the development of advanced, high-throughput, miniaturised devices. The recent advancements in manufacturing graphene-based hybrid composites have demonstrated the promising capabilities of these materials in creating switchable devices,^3–5^ sensors^6–8^ and high performance nanodevices.^9,10^ Fabrication of graphene-based micronano structures through the incorporation of biomolecules has shown significant promise due to the complex morphologies with unique functionalities controlled by the inherent components.

In particular, graphene oxide (GO) has been emerging as a highly attractive candidate for the development of graphene-based nanodevices.^11^ GO is comprised of graphene, an atomic layer of sp^2^-hybridised carbon atoms, functionalised with oxygen-containing functional groups (OCFGs) such as carboxyl, carbonyl, epoxide and hydroxyl groups.^12^ Non-covalent interactions between these OCFGs and biomolecules enable the self-assembly of micro-nanostructures and the interactions between the OCFGs and water molecules render GO hydrophilic, enabling the mass production of these through chemical methods in aqueous media.^13,14^ Graphene-based aerogels have demonstrated unique properties including, high strength, low density and three-dimensional (3D) interconnected structures with macroscale dimensions,^15^ which have been exploited for various applications *e.g.*, wearable sensors,^13,14^ electrodes^16,17^ and adsorbents.^18,19^ Recently a robust method of fabricating graphene-based aerogels has been demonstrated through the self-assembly of graphene oxide (GO) and M13 bacteriophage,^20^ a filamentous virus with a diameter of 6.6 nm and length of 880 nm.^20^ M13 replicates by infecting *Escherichia coli* (*E. coli*), without destroying the host cell (lysogenic cycle) and consists of circular-shaped single-stranded DNA encapsulated by 2700 copies of the pVIII major coat protein, with its terminal areas comprised of minor coat proteins pIII and pVI at its head and pVII and pIX at its tail.^21^

The novel micro-nano GraPhage13 has been fabricated *via* integrating GO and M13 at pH 4.9, yielding an aggregate, where M13 viral strands act as a cross-linker between the GO sheets, enabling its self-assembly. Subsequently, a GraPhage13 hydrogel (GPH) was fabricated, which upon deposition on a supporting substrate in a vacuum, yielded a stable GraPhage13 aerogel (GPA).^20^ The nanofabricated GPA exhibits a number of unique properties, including ultra-high-surface-area (325 m^2^ g^−1^) and low-density (8.8 mg cm^−3^) due to its micro-porous structure as well as being scalable, eco-friendly and of a low cost, rending itself suitable for the incorporation with a broad range of further nanomaterials including nanoparticles,^22^ fluorophores^23^ and polymers.^24^ For instance, Sun *et al.* accomplished the incorporation of carbon nanotubes into the GPA structures, resulting in the production of GPA–CNT. This hybrid material exhibited an electrical conductivity enhancement of up to 30 times when compared to pure GPA.^25^ Furthermore, harnessing the capacity to manipulate the genetic and chemical properties of M13 holds significant potential for altering the characteristics of GPA, such as enhancing its electrical^26–28^ and binding properties.^22,29,30^ These features make GraPhage13 a promising candidate for applications in functional scaffolds, gas filters, energy storage and biological and chemical sensors.^11,15,31,32^

It is therefore paramount to establish the pH range in which GO and M13 can form a dispersion that facilitates their aggregation for GPA self-assembly. This will facilitate the evaluation of its processability and, on a broader scale, the exploration of potential significant applications in aqueous environments characterised by varying pH levels. Herein, a systematic examination was conducted to determine the concentration of ionised groups, p*K*_a_ distribution of the acid groups and the zeta potential of GO, M13 and GPH. The utilisation of an innovative new data analysis technique facilitated the comparison of two distinct experiments, thereby elucidating the relationship between the ionisation of functional groups and surface charge and enabled the investigation of this variation with pH and the determination of its origin. These findings significantly advanced the understanding of the fundamental self-assembly mechanism underlying the GPA fabrication and the specific pH range conducive to GPH assembly. Such insights hold a great significance for further studies of incorporating various nanomaterials into the GPA and exploitation of the GraPhage13 for a wide range of potential applications. This approach could also be for instance, utilized to characterise the interaction of GO with other biomolecules, such as double stranded DNA fragments, proteins and enzymes, to mass produce hydrogels for the manufacture of graphene-based nanodevices for catalysis, dye adsorption and environmental recovery.^11,33–35^

## 2. Results and discussion

### 2.1. Concentration of ionised groups and p*K*_a_

**Fig. 1:**
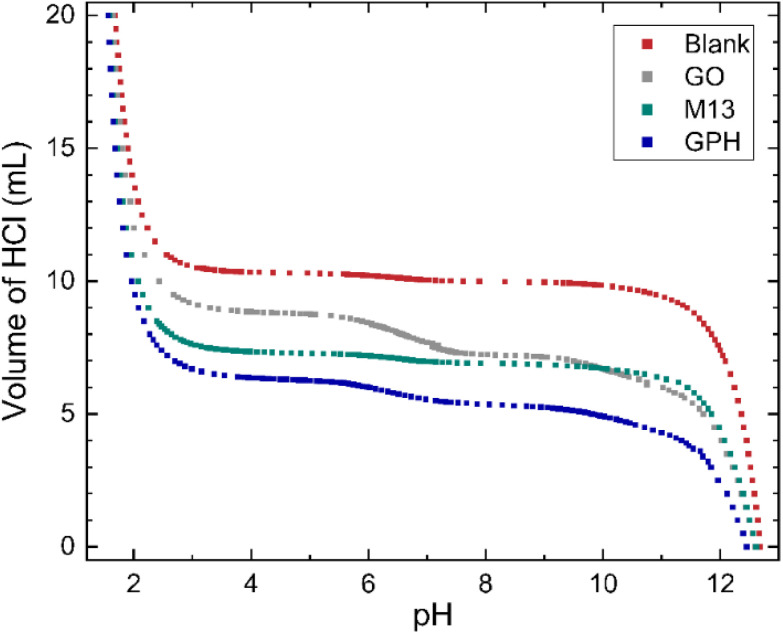
The relationship between pH and volume of 20 mL 50 mM HCl added to 10 mL of titrand 50 mM NaOH. For titrations involving the GO, M13 bacteriophage and GPH, 10 mg of analyte was added.

**Fig. 2:**
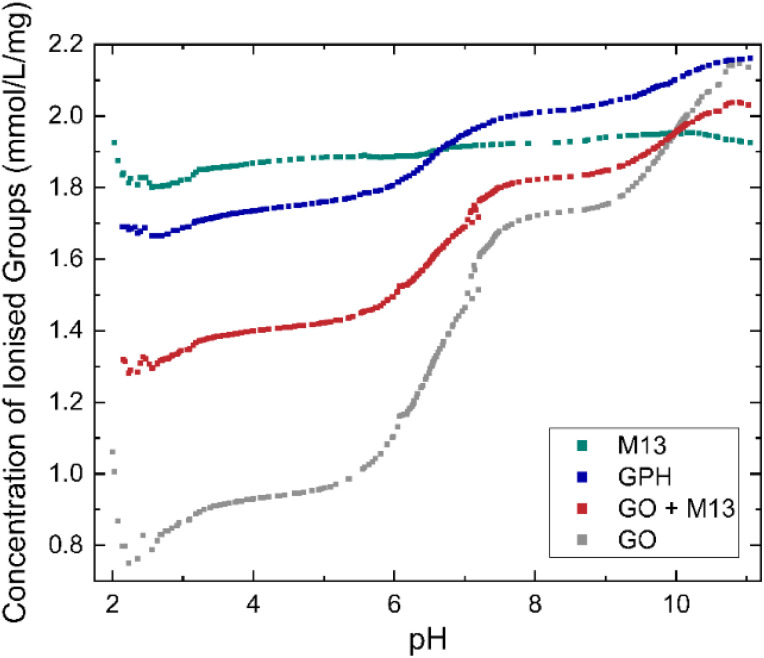
Concentration of ionised groups of GO, M13 bacteriophage, GPH and GO + M13 across a range of pH values.

The relationship between the volume of HCl added to the titrand and its pH is shown in Fig. 1. At extreme pH values a large volume of HCl is required to change the pH, for example the final 5 mL of HCl added to the titrand adjusted the pH by 0.12–0.25. On the other hand, a plateau is observed between pH 3.5–10.5, with 0.7 mL–2.55 mL of HCl (see ‘Methods’) required to generate a significant change. The concentration of ionised groups has been obtained by subtracting the blank titration data from those involving each of the analytes and is given by the difference in the volume of HCl at a particular pH (Fig. 1). The overall relationship between the concentration of ionised groups and pH for GO, M13, GPH and GO + M13 is shown in Fig. 2.

GO shows the greatest variation in the concentration of ionised groups, ranging from 0.75 mmol L^−1^ mg^−1^ at pH 2.2 mmol L^−1^ g^−1^ to 2.15 mmol L^−1^ mg^−1^ at pH 11, with a significant increase between pH 5.5–7.5. This can be explained *via* the reactions leading to its negative surface charge^36^ including firstly the deprotonation of carboxylic groups: C–COOH + H_2_O → C–COO^−^ + H_3_O^+^ and secondly, the deprotonation of enolic and phenolic groups: C=C–OH + H_2_O ↔ C=CO^−^ + H_3_O^+^. As the pH increases the deprotonation equilibrium is shifted to the right-hand side of the reaction, producing a higher concentration of negatively charged carboxylic, enolic and phenolic groups, whilst the increasing concentration of OH^−^ ions neutralises the disassociated protons, leading to the increase in the concentration of ionised groups with pH^36^ whilst the concentration of ionised groups for M13 remains relatively constant, 1.89 ± 0.03 mmol L^−1^ mg^−1^ over the entire pH range (Fig. 2). Therefore, the variation in concentration observed for GPH and GO + M13 can be attributed to the GO component. The difference between the concentration of ionised groups in GPH and GO + M13 decreases as the pH increases, however between pH of 3.1–5.7 and 7.6–9.2 the concentration remains constant at 0.34 mmol L^−1^ mg^−1^ and 0.19 mmol L^−1^ mg^−1^ respectively.

The probability of GO–M13 aggregation at different pH can be determined by the concentration of ionised groups employing Deryaguin–Landau–Verwey–Overbeek (DLVO) theory, which describes how electrostatic force governs colloidal stability. It assumes that electrostatic repulsion and attractive van der Waals are the only electrostatic forces acting on colloids, leading to their dispersion or aggregation within a solution. The electrostatic repulsion originates from the electric double layer, where the particles dispersed in aqueous media gain a negative charge from negative ions adsorbing to its surface, which then attract positive charges and produce the double layer. The magnitude of the electrostatic repulsion depends on the concentration of ions, produced by the dissociation of ionised groups within the solution, whereas the influence of concentration on van der Waals forces is negligible.^37,38^

Consequently, increasing the concentration of ionised groups increases the magnitude of electrostatic repulsion, thus reducing the likelihood of flocculation. Since the concentration of ionised groups for GPH increases with pH, with a particularly rapid increase commencing at pH 6 (Fig. 2), it is plausible that the van der Waals forces overcome the electrostatic repulsion (between the negatively charged amino acids on the surface of M13 and the deprotonated carboxylic groups on GO) between pH 2–6, enabling flocculation of GO and M13. It is hypothesised that the electrostatic interactions occur between the carboxylic groups on the GO with positively charged groups of the N-terminus and K_8_ residues of the M13, following the protonation of the carboxyl groups of E_2_, D_4_, D_5_, E_20_ residues.^39^Fig. 2 indicates the presence of acid groups with varying p*K*_a_.

**Fig. 3:**
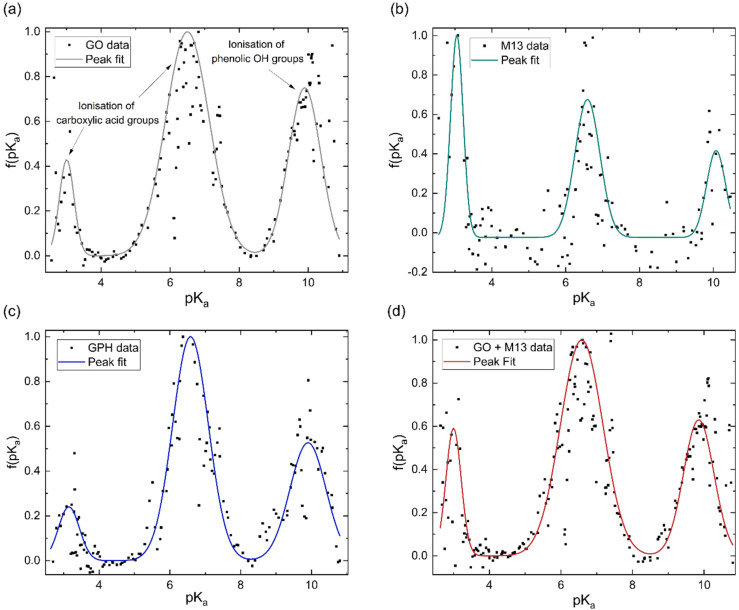
Normalised p*K*_a_ distribution of (a) GO, (b) M13 bacteriophage, (c) GPH and (d) GO + M13. The peaks in (a–d) at p*K*_a_ values of 3.1 and 6.6 are due to the ionisation of carboxylic acid groups and the peak at 9.9 is due to the ionisation of phenolic OH groups (a).

The concentration of groups is subsequently differentiated with respect to pH and Gaussian peaks are fitted to the data to obtain the p*K*_a_ distributions (Fig. 3). Three peaks emerge at nearly the same p*K*_a_ for each analyte, with average positions of 3.1 ± 0.1, 6.6 ± 0.1 and 9.9 ± 0.1. The overall fitted peak positions, widths and intensities are summarised in Table 1.

**Table 1:** p*K*_a_ values, full width half maximum and area for p*K*_a_ distribution of GO, M13, GPH and GO + M13

	Peak 1			Peak 2			Peak 3		
	p*K*_a1_	Width	Area	p*K*_a2_	Width	Area	p*K*_a3_	Width	Area
GO	3.0 ± 0.3	0.5 ± 0.8	0.2 ± 0.3	6.5 ± 0.2	1.5 ± 0.5	1.5 ± 0.5	9.9 ± 0.3	1.1 ± 0.7	0.8 ± 0.5
M13	3.05 ± 0.04	0.40 ± 0.09	0.45 ± 0.09	6.59 ± 0.07	0.8 ± 0.2	0.6 ± 0.1	10.1 ± 0.1	0.6 ± 0.3	0.3 ± 0.1
GPH	3.1 ± 0.1	0.7 ± 0.3	0.19 ± 0.06	6.58 ± 0.03	1.2 ± 0.1	1.31 ± 0.08	9.9 ± 0.1	1.2 ± 0.2	0.7 ± 0.1
GO + M13	3.0 ± 0.2	0.5 ± 0.4	0.3 ± 0.2	6.6 ± 0.1	1.4 ± 0.4	1.4 ± 0.3	9.9 ± 0.2	1.0 ± 0.6	0.7 ± 0.3

It has been previously noted that three peaks are observed in the p*K*_a_ distribution of GO, with values of 4.3 and 6.6, originating from the ionisation of carboxylic acid groups and the peak at 9.9 corresponding to the ionisation of a phenolic OH group.^40^

The presence of these functional groups therefore, account for the peaks observed for GO, GPH and GO + M13 in Fig. 3a–d. The difference between the p*K*_a_ of the first peak (3.1 ± 0.1) in comparison to the value reported in ref. 40 for GO is likely due to the over-data-smoothing at extreme pH values. Table 1 demonstrates that not only are the p*K*_a_ values of GPH and GO + M13 the same but also that their widths and intensities match within error of the peak fit. This indicates that there are no new ionised functional groups generated from the GPH interaction, and therefore GPH exhibits dispersibility over a similar pH range as GO. The known p*K*_a_ values for the M13 bacteriophage are given by Passaretti *et al.*^41^ While the M13 has the same p*K*_a_ values as GO, GPH and GO + M13 (Fig. 3b), a higher signal-to-noise ratio hinders the accuracy of the peak fitting and indicates that there may be peaks which are not detected (Fig. 3b). A second peak fit, the results of which are shown in Table 2, demonstrates the inaccuracy of fitting peaks according to the known p*K*_a_ values of M13.

**Table 2:** p*K*_a_ values of pVIII amino acids contributing towards the surface charge of M13 bacteriophage, obtained through peak fitting to the known p*K*_a_ values of M13.^41^ The K_8_ residue is not included as its p*K*_a_ is beyond the measured values

pVIII amino acid	Known p*K*_a_	Measured p*K*_a_	Peak width	Peak area
A1	8.62	8.9 ± 0.3	0.7 ± 0.8	0.2 ± 0.2
E2	3.45	3.4 ± 0.9	0 ± 2	0 ± 1
D4	3.11	3.0 ± 0.3	0.4 ± 0.4	0.4 ± 0.9
D5	4.02	3.9 ± 0.5	0 ± 1	0.1 ± 0.3
E20	5.21	5.7 ± 0.2	0.5 ± 0.2	0.3 ± 0.2

This could be due to these peaks having low intensity or overlap with further peaks demonstrating similar p*K*_a_ values or the effect of interactions due to the presence of the electric double layer, HCl and NaOH.^36,42,43^ Furthermore, for p*K*_a_ values of less than 2.7–3.8 and more than 10–11, the values are unlikely to be correct due to the inaccuracy of the pH measurements. The Nernst equation, which relates the electrical potential across the electrode to the pH, breaks down at extreme pH values and therefore, a peak due to the presence of the K8 residue, which has a p*K*_a_ of 11.56, cannot be detected.^41,44^ These limitations, therefore, do not enable the p*K*_a_ of specific M13 residues to be identified. This may also impact the comparison of p*K*_a_ values of GPH and GO + M13, as there may be variations in the functional groups as a result of the interaction between GO and M13 that have not been observed.

### 2.2. Zeta potential

**Fig. 4:**
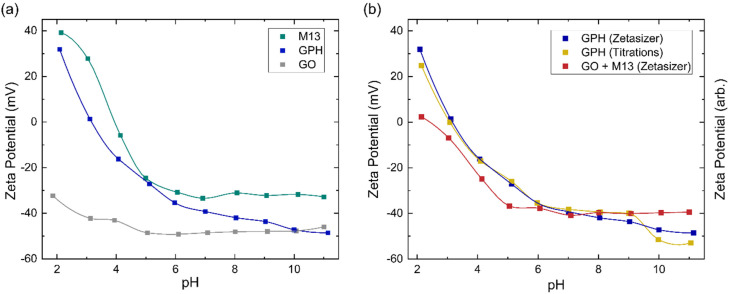
(a) Zeta potential of GO, M13 bacteriophage and GPH. (b) Comparison of zeta potential measurements of GPH to the addition of the individual GO and M13 (GO + M13) along with the zeta potential trend of GPH. The trend lines are generated *via* interpolation.

The zeta potential measurements for GO, M13 and GPH between pH 2–11 are shown in Fig. 4a. The zeta potential of GO is found to exhibit the least variation over this pH range, measuring −32 ± 1 mV at pH 1.9 and −46 ± 1 mV at pH 11.0. M13 shows a larger variation, decreasing from 39.2 ± 0.7 mV at pH 2.1 to −24.6 ± 0.8 mV at pH 5.0, before plateauing at −32.0 ± 0.4 mV between pH 6.1–11.0. GPH demonstrates the widest range, spanning from 31.9 ± 0.1 mV at pH 2.1 to −48.6 ± 0.4 mV at pH 11.1.

Fig. 4a shows that the zeta potential of GO remains below −30 mV. The zeta potential, *ζ*, is the electrical potential at the slipping plane, which provides the magnitude of the electrostatic repulsion between adjacent colloids.^45^ Colloidal dispersions are generally found to be stable when |*ζ*| > 30 mV, due to strong electrostatic repulsion between like charges. For the colloids to flocculate the electrostatic repulsion must be overcome by the attractive van der Waals forces,^46^ that is to achieve |*ζ*| < 30 mV,^47^ which would result in GO forming a stable colloid which does not aggregate between pH 2–11. As the pH of 1 mg mL^−1^ GO was measured to be 2.4, NaOH was added to GO to increase the pH to 3–11. The NaOH acted as a hydrogenating agent and reduced GO to activated graphene, decreasing its sheet size to form a more stable dispersion. This leads to an improved colloidal stability and therefore, decreases the probability of flocculation.^48^ HCl was added to measure GO at pH 2, however only a low concentration was required to adjust the pH such that very few protons are disassociated from their acidic functional groups, and therefore the zeta potential remains in the stable colloid range. As the pH increases the zeta potential turns more negative due to the increased concentration of negatively charged ionised groups.^36^ A small increase in the zeta potential is observed at high pH, due the high concentration of OH^−^ ions causing double layer compression.^40^

Zeta potential measurements for M13 show that the bacteriophage is a stable colloid at pH < 3 and pH > 6, which flocculates between pH 3–6. The surface charge of M13 is a result of a protonation and deprotonation of the A1, E2, D4, D5, K8 and E20 residues and the amino group at the N-terminus. Since these residues have differing p*K*_a_ values, their charge and therefore the surface charge of M13 are dependent on the pH of the chemical environment.^41,49^ The zeta potential of both M13 and GPH decreases as the pH increases until pH 6, whereafter the zeta potential of M13 plateaus while GPH continues to decrease as the pH increases, eventually overlapping with GO at pH > 10. This suggests that below pH 6 the main contributor towards surface charge of GPH is the M13. Above pH 6, where the chemical environment becomes more basic, there is a higher probability of GO deprotonation, producing ionised groups, which contribute towards the GPH's surface charge. Having established from the concentration of ionised groups that the aggregation of GO and M13 is only possible between pH 2–6, it can be concluded that M13 is the primary contributor to the surface charge of GPH.

Fig. 4a further demonstrates that aggregation between GO and M13 occurs between 2.1 ± 0.4 > pH > 5.4 ± 0.9, in agreement with the previously determined range of pH 2–6. To assess the implications of this on the processability of GPA, both the forces involved in aggregation and the likelihood of flocculation need to be examined. The probability of aggregation can be analysed *via* the sticking probability *P*_s_ = *P*_1_ 10^−|pH–PZC|^, where *P*_1_ is the sticking probability of an individual particle and PZC is the point of zero charge. The PZC corresponds to the pH at which a particle has zero surface charge such that the electrostatic repulsion is minimised. The optimal pH for aggregation is slightly above the PZC. As the pH deviates further from the PZC the electrostatic repulsion increases, reducing the probability of flocculation.^50^ Since the GPH aggregation is possible at 2.1 ± 0.4 > pH > 5.4 ± 0.9, it can be estimated that the PZC for GPH is roughly an average pH value of this range, 3.8 ± 0.5. Previously, it was determined that the optimal pH for GPH aggregation is 4.9,^20^ which is in agreement with the discussion establishing that the optimal pH should be slightly above the PZC.

A comparison between the zeta potential of GPH and the addition of the GO and M13 zeta potential measurements (GO + M13) is shown in Fig. 4b. Zeta potential of GO + M13 is, on average, 34 ± 3 mV lower than GPH for all pH values. The difference between these trends confirms that the interaction between GO and M13 produces an aggregate with a lower surface charge than the sum of the surface charges of GO and M13, which is beneficial to the fabrication of the micronano sponge. Fig. 4b illustrates the difference between GPH zeta potential measurements and the trend of the zeta potential from the titration data. Zeta potential can be determined by multiplying the concentration of ionised groups with the charge on that group. However, since the electrochemical active surface area is unknown only the trends of the zeta potentials can be compared. As the surface charge is proportional to the zeta potential, the concentration of ionised groups was multiplied by the measured GPH zeta potential and scaled such that the trends could be compared. The significant overlap between titration and Zetaziser measurements imply that the ionised groups almost completely determine the variation of surface charge of GPH with pH.

## 3. Conclusions

The relationship between pH and the aggregation of graphene oxide–M13 bacteriophage, which in turn enables the self-assembly of GraPhage13 aerogels, has been investigated through pH titrations and zeta potential measurements. The concentration of ionised groups for M13 bacteriophage remains constant, whereas for GO it is dependent on the direction of the deprotonation, which is heavily influenced by its chemical environment. Furthermore, it has been shown that the concentration of ionised groups to be higher for GPH than the addition of GO and M13. Using DLVO theory, it has been determined that the GO–M13 aggregation occurs between 2 < pH < 6. The p*K*_a_ values for GO were found to be 3.1, 6.6 and 9.9, corresponding to the ionisation of carboxylic and phenolic OH groups, and no new functional groups were found to be formed as a result of the interaction between GO and M13. Zeta potential measurements demonstrate that GO remains stable across a wide pH range of 2–11, whilst M13 forms unstable dispersions and flocculates between pH 3–6. Similar to the concentration of ionised groups, aggregation of GO and M13 occurs at 2.1 ± 0.4 > pH 5.4 ± 0.9. Employing DLVO theory and sticking probabilities the optimal pH was established to be slightly above the estimated value for the point of zero charge at 3.8 ± 0.5, in agreement with previous research showing the optimal pH for aggregation of pH 4.9.

Observing the dispersibility of GPH over a wide range of pH values enabled determining the origin of the GPH surface charge and the pH range in GPA fabrication, providing an important insight into the underpinning mechanism behind the self-assembly of GraPhage13 aerogels. By employing an innovative technique, a comparative analysis was conducted between the zeta potential trends through titrations and zeta potential measurements. The similarity in zeta potential trends provides evidence that the surface charge of GPH arises from presence of ionised groups and electrostatic interactions between them. More specifically, it can be attributed to interactions between the carboxylic groups of GO with positively charged N-terminus and the K8 residue of M13 following the protonation of the E20 residue. These interactions play an essential role in facilitating the self-assembly of GPAs. Furthermore, within the pH range in which the aggregation occurs, the main contributor to the surface charge was determined to be the M13 bacteriophage. The self-assembly of GraPhage13 depends on reducing the electrostatic repulsion between the negatively charged amino acids of M13 and the deprotonated carboxylic groups of GO, which can be achieved by lowering the concentration of ionised groups. Combining GO and M13 in a chemical environment of pH 2–6 reduces the likelihood of the deprotonation of the carboxylic groups in GO, enabling the generation of GPH and the subsequent fabrication of GPA.

This novel investigation, utilising a new analytical technique to compare the concentration of ionized groups and surface charge across different pH levels, has yielded profound insights into the dispersibility of GraPhage13 and the origin of the ionised groups responsible for its surface charge and self-assembly. This enhances our understanding of its processability, thereby providing guidance for the future integration of various nanomaterials including nanoparticles, polymers and fluorophores into the aerogel structure. This opens up possibilities for the development of graphene-based nanodevices for a broad range of applications including micronano filters, functional scaffolds and miniaturised sensors.

## 4. Materials and methods

### 4.1. Optimised propagation and purification of M13 bacteriophage

The propagation of M13 bacteriophage (New England Biolabs, UK) was carried out using One Shot TOP10F’ Chemically Competent *E. coli* (Thermo Fisher Scientific). *E. coli* cells were first cultivated by inoculating nutrient broth (NB) agar plates and incubating them overnight at 37 °C. The *E. coli* was then transferred from the Petri dishes into 50 mL falcon tubes, containing Nutrient Broth (NB) and tetracycline in ethanol to a final concentration of 5 μg mL^−1^, which were incubated overnight in a shaker incubator at 37 °C, 150 rpm. This solution is directly deposited into the M13 propagation process. The protocol for propagating and purifying M13 bacteriophage is based on Passaretti *et al.*^51^ Briefly, M13 was incubated overnight in NB (Sigma) with the *E. coli* – NB – tetracycline solution and tetracycline in ethanol (Sigma), to a final concentration of 5 μg mL^−1^. The solution was centrifuged twice (Beckman Coulter, JLA 10.5), combined with a mixture of 25% polyethylene glycol (PEG) 6000 and 2.5 M NaCl and left to stir on ice for 90 minutes. Centrifuging produced a white pellet, which was resuspended in deionised water (DIW), deposited in 1.5 mL tubes and centrifuged in a microcentrifuge (SciSpin MICRO). The supernatant was transferred to new microcentrifuge tubes, PEG + NaCl was added and left on ice for 60 minutes. These subsequently underwent a final microcentrifuge, producing a white pellet of M13 which was resuspended in DIW.

### 4.2. UV-Vis spectroscopy

The concentration of M13 in DIW was determined using a UV-Vis spectrophotometer (Aligent Cary 60 UV-Vis), with a 1 cm light path quartz cuvette. A spectrum of DIW was first taken as a baseline. Prior to sample analysis, they are transferred into a 1.5 mL centrifuge tube and placed in an orbital shaker for 1 minute to ensure the uniformity of M13 in DIW. The concentration of M13 was then determined from the resulting spectrum using the Beer–Lambert Law and an extinction coefficient of 3.84 cm^2^ mg^−1^ at 269 nm. The viability of the M13 bacteriophage was confirmed through the presence of specific characteristics present in the spectrum (Fig. S3†) including the absorbance peak at 269 nm (*A*_269_), a local minimum absorbance at 245 nm (*A*_245_) and a baseline at 350 nm (*A*_350_). The produced phages are pure and viable if the *A*_269_/*A*_245_ ratio is ∼1.37 and the *A*_350_/*A*_269_ is ∼0.02.^52^

### 4.3. Titrations

Titrations were performed using a pH meter (Mettler Toledo FiveEasy), calibrated with standard buffer solutions of pH 4, 7 and 10 (Fisher Chemical). Graphene Oxide (GO) with concentration of 5 mg mL^−1^ was obtained from Graphene Supermarket (SKU-HCGO-W-175ML) with a composition of 79% carbon and 20% oxygen, flake size of 0.5–5 μm and at least 60% of the GO with a thickness of one atomic layer.^53^ The M13 was prepared as described above and diluted to a concentration of 5 mg mL^−1^. Solutions of 50 mM HCl and 50 mM NaOH were produced by diluting concentrated HCl (Merck Life Sciences Ltd, 30721-1L-M) and dissolving NaOH pellets (Scientific Laboratory Supplies Ltd, 71690-500G) in DIW. To confirm the concentrations the pH of these solutions was measured, assuming the HCl and NaOH were the only source of H^+^ ions, 50 mM HCl and 50 mM NaOH corresponding to pH 1.3 and pH 12.7, respectively. The titrations were carried out within a 50 mL beaker with a stirrer bar placed on a magnetic stirring plate. During the titrations the stirrer bar was continually rotated to ensure the pH was uniform throughout the solution. The sensor at the end of the pH probe was held above the stirrer bar within the solution. As pH is temperature-dependent, all pH readings were taken in a temperature-controlled environment to reduce error. Blank titrations were first taken as a baseline, with 20 mL of HCl being added incrementally to 10 mL of NaOH. According to the stoichiometry, HCl and NaOH neutralises to pH 7 when equal concentrations and volumes are combined. Therefore, the addition of 20 mL of HCl to 10 mL of NaOH allowed pH changes in both the acid and alkali ranges to be observed. 10 mL of NaOH was deposited into the beaker. Varying volumes of HCl were then added to the NaOH with a pipette and the total volume of HCl and the pH was recorded. The volume of HCl added was dependent on the required pH. At the extrema of the pH scale, the addition of 1000 μL produced a change in pH of between 0.01–0.1 whereas between pH 4–5 and pH 8–9, 10 μL of HCl altered the pH by up to 0.54. Therefore, the increments in which HCl was added to NaOH varied depending on the measured pH. For the GO and M13 titrations, 8 mL of NaOH was combined with 2 mL (10 mg) of GO and M13 respectively, prior to the addition of HCl. Similarly, the GPH titrations involved combining 6 mL of NaOH with 2 mL of GO and 2 mL of M13. Each titration (blank, GO, M13 and GPH) was repeated three times.

### 4.4. Data processing

A new optimised data analysis protocol which accurately and appropriately yields the p*K*_a_ distribution from a designed titration experiment was developed. Firstly, the measured pH value was set as abscissa and the input amount of acid as ordinate, since it represented the difference in the amounts of required acid between with and without (the blank titration as reference) the sample to reach a pH value of interest. Next, a time series representative of the pH values was generated from the amount of added acid and times (now specified by the pH). Since the discrete data points of the sample and the reference did not have the same set of values on abscissa (pH), it was not possible to directly obtain the difference in the amount of acid from the data points. Due to the logarithmic nature of pH, fitting polynomial curves between successive data points (interpolation) was not applicable at extreme pH values, where these scarcely changed when adding acid, with the linear term coefficient approaching infinity. Time series therefore introduced a viable solution. Subsequently, the difference between the time series of the sample and the reference was obtained and the abscissa of times was converted back to pH, enabling plotting the difference as discrete points, representing the concentration of analysed groups for each analyte. To compare the concentration of ionised groups for each analyte, the volume of HCl was converted into moles and divided by the quantity of NaOH within the titrand, and subsequently divided by the weight of analyte in the titrand. Finally, the first derivative of the difference as a time series was calculated to obtain the p*K*_a_ distribution. The abscissa of times was converted back to p*K*_a_ after the differentiation. The optimised data analysis method was performed using built-in functions *via* Wolfram Mathematica.

### 4.5. Scanning electron microscopy and energy dispersive X-ray spectroscopy characterization

GPA morphology was characterised using a Hitachi SU5000 scanning electron microscope (SEM) (Fig. S1†). Due to the insulating nature of GPAs, SEM images were acquired at a low voltage of 0.5 kV to minimise charging effects. The elemental composition was further analysed *via* energy-dispersive X-ray spectroscopy (EDX). The EDX spectra (Fig. S2†) were obtained at 15 kV using a Hitachi TM3030 microscope equipped with Oxford Instruments Swift ID.

### 4.6. Zeta potential

Zeta potential measurements were taken using a Malvern Zetasizer Ultra. Solutions between pH 2–11 were produced with NaOH and HCl. GO and/or M13 were added to a final concentration of 1 mg mL^−1^ and the pH was measured. The solutions were deposited in folded capillary zeta cells and placed in the Zetasizer for analysis.

## Author contributions

PGO and YS conceptualized the study. KS and PP carried out the experimental acquisition and analyses and validated the results. PGO and HW were responsible for funding acquisition and resources provision. PGO supervised and administered the study, curated the data and overviewed the developed methodology. All authors contributed to writing the original draft and PGO, YS and KS reviewed and edited the final draft.

## Conflicts of interest

There are no conflicts to declare.

## Supplementary Material

NR-015-D3NR00778B-s001

## References

[cit1] Maiti D., Tong X., Mou X., Yang K. (2019). Carbon-Based Nanomaterials for Biomedical Applications: A Recent Study. Front. Pharmacol..

[cit2] Rauti R., Musto M., Bosi S., Prato M., Ballerini L. (2019). Properties and behavior of carbon nanomaterials when interfacing neuronal cells: How far have we come?. Carbon.

[cit3] Pal K., Mohan M. L. N. M., Foley M., Ahmed W. (2018). Emerging assembly of ZnO-nanowires/graphene dispersed liquid crystal for switchable device modulation. Org. Electron..

[cit4] Pal K. (2021). *et al.*, Cutting edge development on graphene derivatives modified by liquid crystal and CdS/TiO2 hybrid matrix: optoelectronics and biotechnological aspects. Crit. Rev. Solid State Mater. Sci..

[cit5] Si A., Kyzas G. Z., Pal K., de Souza Jr. F. G. (2021). Graphene functionalized hybrid nanomaterials for industrial-scale applications: A systematic review. J. Mol. Struct..

[cit6] Arfin T. , Functional graphene-based nanodevices: emerging diagnostic tool, in Nanomaterials in Diagnostic Tools and Devices, ed. S. Kanchi and D. Sharma, Elsevier, 2020, ch. 3, pp. 85–112

[cit7] Cordaro A., Neri G., Sciortino M. T., Scala A., Piperno A. (2020). Graphene-Based Strategies in Liquid Biopsy and in Viral Diseases Diagnosis. Nanomaterials.

[cit8] Soren S., Chakroborty S., Pal K. (2022). Enhanced in tunning of photochemical and electrochemical responses of inorganic metal oxide nanoparticles via rGO frameworks (MO/rGO): A comprehensive review. Mater. Sci. Eng., B.

[cit9] Pham K. D., Nguyen C. Q., Nguyen C. V., Cuong P. V., Hieu N. V. (2021). Two-dimensional van der Waals graphene/transition metal nitride heterostructures as promising high-performance nanodevices. New J. Chem..

[cit10] Fried J. P., Swett J. L., Bian X., Mol J. A. (2018). Challenges in fabricating graphene nanodevices for electronic DNA sequencing. MRS Commun..

[cit11] Passaretti P. (2022). Graphene Oxide and Biomolecules for the Production of Functional 3D Graphene-Based Materials. Front. Mol. Biosci..

[cit12] Park S., Ruoff R. S. (2009). Chemical methods for the production of graphenes. Nat. Nanotechnol..

[cit13] Zhang F. (2022). *et al.*, A highly accurate flexible sensor system for human blood pressure and heart rate monitoring based on graphene/sponge. RSC Adv..

[cit14] Zhang D. (2022). *et al.*, Multifunctional Superelastic Graphene-Based Thermoelectric Sponges for Wearable and Thermal Management Devices. Nano Lett..

[cit15] Chabot V., Higgins D., Yu A., Xiao X., Chen Z., Zhang J. (2014). A review of graphene and graphene oxide sponge: material synthesis and applications to energy and the environment. Energy Environ. Sci..

[cit16] Duinslaeger N., Radjenovic J. (2022). Electrochemical degradation of per- and polyfluoroalkyl substances (PFAS) using low-cost graphene sponge electrodes. Water Res..

[cit17] Ormeno-Cano N., Radjenovic J. (2022). Electrochemical degradation of antibiotics using flow-through graphene sponge electrodes. J. Hazard. Mater..

[cit18] Xu P., Rahmani F., Chiew Y. C. (2022). Adsorption and diffusion of methane and light gases in 3D nano-porous graphene sponge. Mol. Simul..

[cit19] Maimaiti T. (2022). *et al.*, Magnetic Fe3O4/TiO2/graphene sponge for the adsorption of methylene blue in aqueous solution. Diamond Relat. Mater..

[cit20] Passaretti P. (2019). *et al.*, Multifunctional graphene oxide-bacteriophage based porous three-dimensional micro-nanocomposites. Nanoscale.

[cit21] Han S. M. (2022). *et al.*, M13 Bacteriophage-Based Bio-nano Systems for Bioapplication. BioChip J..

[cit22] Hou J., Xu Y., Sun S., Zhong X., Yang C.-T., Zhou X. (2023). Gold nanoparticles-decorated M13 phage SPR probe for dual detection of antigen biomarkers in serum. Sens. Actuators, B.

[cit23] Huang S. (2019). *et al.*, M13 Virus-Based Framework for High Fluorescence Enhancement. Small.

[cit24] Dong X., Pan P., Ye J.-J., Zhang Q.-L., Zhang X.-Z. (2022). Hybrid M13 bacteriophage-based vaccine platform for personalized cancer immunotherapy. Biomaterials.

[cit25] Sun Y. (2020). *et al.*, Nanomechanics of graphene oxide-bacteriophage based self-assembled porous composites. Sci. Rep..

[cit26] Kim H. (2021). *et al.*, M13 Virus Triboelectricity and Energy Harvesting. Nano Lett..

[cit27] Park I. W. (2020). *et al.*, Recent Developments and Prospects of M13- Bacteriophage Based Piezoelectric Energy Harvesting Devices. Nanomaterials.

[cit28] Han J. (2021). *et al.*, Genetic Manipulation of M13 Bacteriophage for Enhancing the Efficiency of Virus-Inoculated Perovskite Solar Cells with a Certified Efficiency of 22.3%. Adv. Energy Mater..

[cit29] Bortot B. (2022). *et al.*, Advanced photodynamic therapy with an engineered M13 phage targeting EGFR: Mitochondrial localization and autophagy induction in ovarian cancer cell lines. Free Radicals Biol. Med..

[cit30] Wang R., Li H.-D., Cao Y., Wang Z.-Y., Yang T., Wang J.-H. (2023). M13 phage: a versatile building block for a highly specific analysis platform. Anal. Bioanal. Chem..

[cit31] Tung T. T. (2017). *et al.*, Recent Advances in Sensing Applications of Graphene Assemblies and Their Composites. Adv. Funct. Mater..

[cit32] Thakur A. (2022). Graphene aerogel based energy storage materials – A review. Mater. Today: Proc..

[cit33] Xu W. (2019). *et al.*, Graphene oxide enabled long-term enzymatic transesterification in an anhydrous gas flux. Nat. Commun..

[cit34] Xu Y., Wu Q., Sun Y., Bai H., Shi G. (2010). Three-Dimensional Self-Assembly of Graphene Oxide and DNA into Multifunctional Hydrogels. ACS Nano.

[cit35] Ardini M. (2016). *et al.*, Supramolecular self-assembly of graphene oxide and metal nanoparticles into stacked multilayers by means of a multitasking protein ring. Nanoscale.

[cit36] Szabó T., Tombácz E., Illés E., Dékány I. (2006). Enhanced acidity and pH-dependent surface charge characterization of successively oxidized graphite oxides. Carbon.

[cit37] Park S.-J. and Seo M.-K., Intermolecular Force, in Interface Science and Technology, ed. S.-J. Park and M.-K. Seo, Elsevier, 2011, ch. 1, vol. 18, pp. 1–57

[cit38] Hallett J. E., Gillespie D. A. J., Richardson R. M., Bartlett P. (2018). Charge regulation of nonpolar colloids. Soft Matter.

[cit39] Passaretti P. , M13 Bacteriophage: A Nanotool for the Fabrication of Novel Self-assembled Nanostructures, PhD Thesis in Nanotechnology, Chemical Engineering, University of Birmingham, 2019

[cit40] Konkena B., Vasudevan S. (2012). Understanding Aqueous Dispersibility of Graphene Oxide and Reduced Graphene Oxide through pKa Measurements. J. Phys. Chem. Lett..

[cit41] Passaretti P., Sun Y., Dafforn T. R., Oppenheimer P. G. (2020). Determination and characterisation of the surface charge properties of the bacteriophage M13 to assist bio-nanoengineering. RSC Adv..

[cit42] Orth E. S. (2016). *et al.*, pKa determination of graphene-like materials: Validating chemical functionalization. J. Colloid Interface Sci..

[cit43] Dimiev A. M., Alemany L. B., Tour J. M. (2013). Graphene Oxide. Origin of Acidity, Its Instability in Water, and a New Dynamic Structural Model. ACS Nano.

[cit44] Gameiro P., Reis S., Lima J. L. F. C., de Castro B. (2000). Calibration of pH glass electrodes by direct strong acid/strong base titrations under dilute conditions. Anal. Chim. Acta.

[cit45] Adair J. H. , Suvacı E. and Sindel J., Surface and Colloid Chemistry, 2001

[cit46] Lozano M. V. , Santander-Ortega M. J. and Alonso M. J., In vitro relevant information for the assessment of nanoparticles for oral drug administration, in Nanotechnology for Oral Drug Delivery, ed. J. P. Martins and H. A. Santos, Academic Press, 2020, ch. 14, pp. 419–458

[cit47] Joseph E. and Singhvi G., Multifunctional nanocrystals for cancer therapy: a potential nanocarrier, in Nanomaterials for Drug Delivery and Therapy, ed. A. M. Grumezescu, William Andrew Publishing, 2019, ch. 4, pp. 91–116

[cit48] Kashyap S., Mishra S., Behera S. K. (2014). Aqueous Colloidal Stability of Graphene Oxide and Chemically Converted Graphene. J. Nanopart..

[cit49] Putra B. R. (2020). *et al.*, Bacteriophage M13 Aggregation on a Microhole Poly(ethylene terephthalate) Substrate Produces an Anionic Current Rectifier: Sensitivity toward Anionic versus Cationic Guests. ACS Appl. Bio Mater..

[cit50] Xiong Y., Liu X., Xiong H. (2021). Aggregation modeling of the influence of pH on the aggregation of variably charged nanoparticles. Sci. Rep..

[cit51] Passaretti P., Khan I., Dafforn T. R., Goldberg Oppenheimer P. (2020). Improvements in the production of purified M13 bacteriophage bio-nanoparticle. Sci. Rep..

[cit52] Morag O., Sgourakis N. G., Abramov G., Goldbourt A. (2018). Filamentous Bacteriophage Viruses: Preparation, Magic-Angle Spinning Solid-State NMR Experiments, and Structure Determination. Methods Mol. Biol..

[cit53] Graphene Supermarket. Graphene Oxide. Graphene Supermarket. https://cdn.shopify.com/s/files/1/0572/0188/5393/files/Graphene_Oxide-Data-Sheet-Graphene-Supermarket.pdf?v=1626799704

