# Supplementary material for: Optimisation of GraPhage13 macro-dispersibility via understanding the pH-dependent ionisation during self-assembly: towards the manufacture of graphene-based nanodevices
Source: Nanoscale. 2023 Jul 25;15(32):13304–12. doi: 10.1039/d3nr00778b (PMC10433945; doi:10.1039/d3nr00778b)
Supplement: NR-015-D3NR00778B-s001 [file NR-015-D3NR00778B-s001.pdf]

Electronic Supplementary Material (ESI) for Nanoscale.
This journal is © The Royal Society of Chemistry 2023

# - Supporting Information -

Optimisation of GraPhage13 Macro-Dispersibility *via* Understanding the pH-Dependent Ionisation During Self-assembly: Towards the Manufacture of Graphene-based Nanodevices

Kate Stokes [1], Yiwei Sun [1], Paolo Passaretti [2], Henry White, [3] Pola Goldberg Oppenheimer [1,4,]*

[1] School of Chemical Engineering, Advanced Nanomaterials Structures and Applications Laboratories, College of Engineering and Physical Sciences, University of Birmingham, Edgbaston, Birmingham, B15 2TT, UK
[2] Institute of Cancer and Genomic Sciences, College of Medical and Dental Sciences, University of Birmingham, B15 2TT, UK
[3] BAE-Systems, Air Sector, Buckingham House, FPC 267, Filton, Bristol, UK
[4] Healthcare Technologies Institute, Institute of Translational Medicine, Mindelsohn Way, Birmingham, B15 2TH, UK

S1. SEM Characterisation:

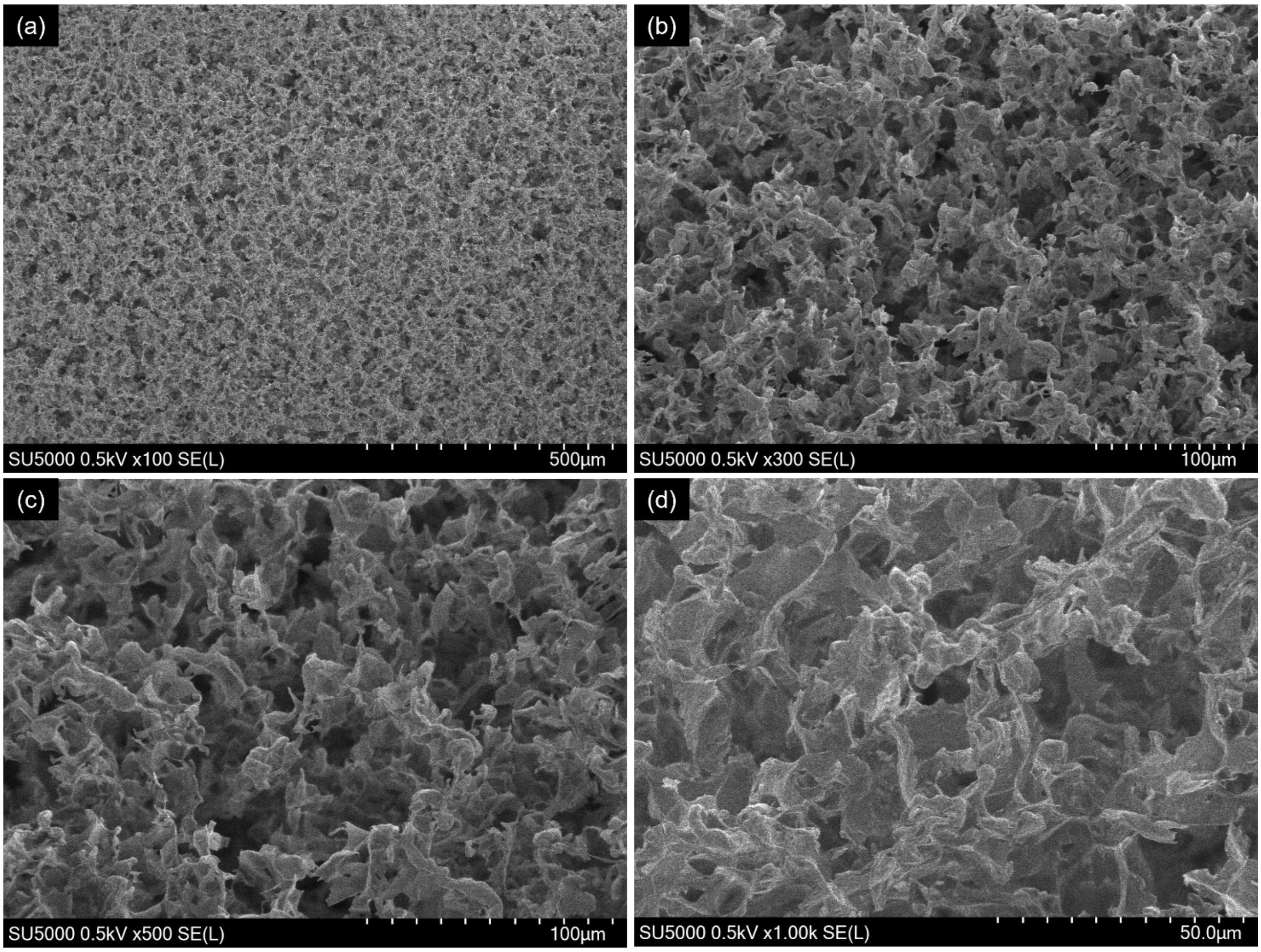

Figure S1. SEM images of GraPhage13 aerogels (GPA) at **(a)** 100x, **(b)** 300x, **(c)** 500x and **(d)** 1000x magnifications.

S2. EDX Analysis:

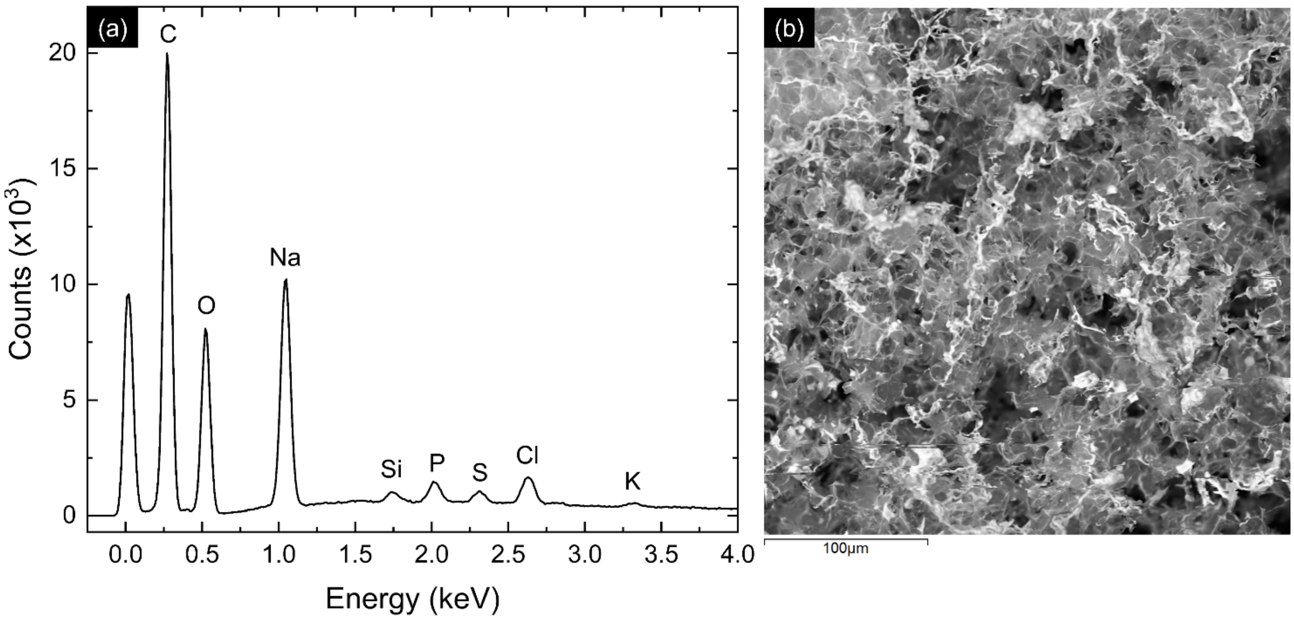

Figure S2. (a) EDX of GraPhage13 aerogel, generated from a map scan over the area shown in the SEM image **(b)**.

S3. UV-Vis Analysis:

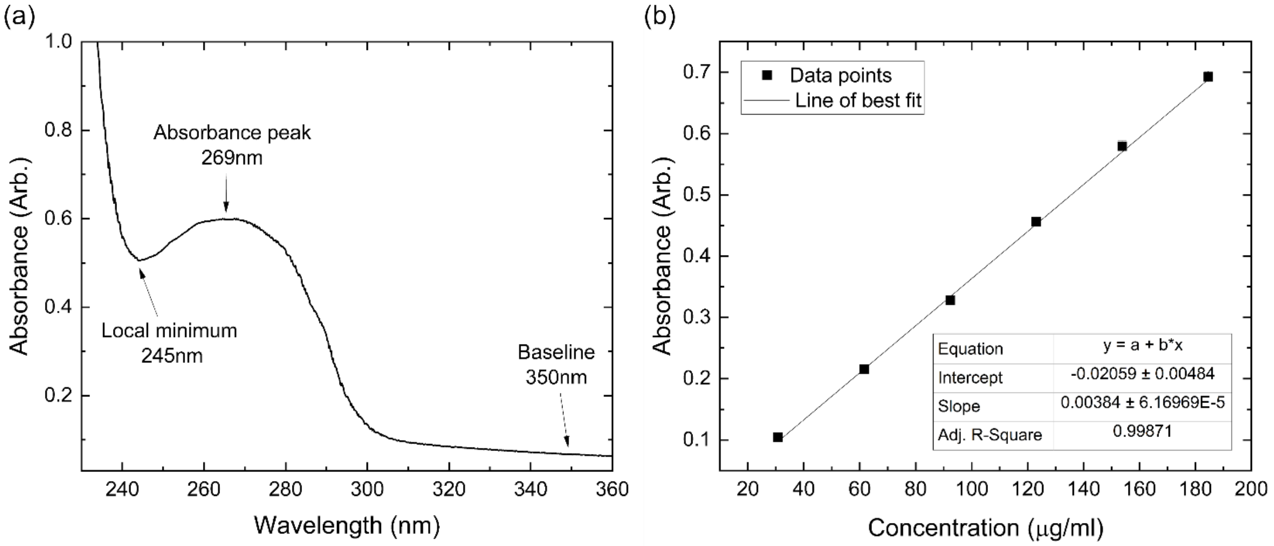

Figure S3. (a) Representative UV-Vis spectrum of M13 bacteriophage with the characteristic features labelled. The absorbance at 269nm was measured at various M13 concentrations, generating the corresponding **(b)** calibration curve.
